# On Seizing the Source: Toward a Phenomenology of Religious Violence

**DOI:** 10.1080/09672559.2016.1284785

**Published:** 2017-02-21

**Authors:** Michael Staudigl

**Affiliations:** ^a^ Department for Philosophy, University of Vienna, Vienna, Austria

**Keywords:** Phenomenology, religion, violence, Paul Ricœur, Jean-Luc Marion

## Abstract

In this paper I argue that we need to analyze ‘religious violence’ in the ‘post-secular context’ in a twofold way: rather than simply viewing it in terms of mere irrationality, senselessness, atavism, or monstrosity – terms which, as we witness today on an immense scale, are strongly endorsed by the contemporary theater of cruelty committed in the name of religion – we also need to understand it in terms of an ‘originary supplement’ of ‘disengaged reason’. In order to confront its specificity beyond traditional explanations of violence, I propose an integrated phenomenological account of religion that traces the phenomenality of religion in terms of a correlation between the originary givenness of transcendence and capable man’s creative capacities to respond to it. Following Ricœur, I discuss ‘religious violence’ in terms of a monopolizing appropriation of the originary source of givenness that conflates man’s freedom to poetically respond to the appeal of the foundational with the surreptitiously claimed sovereignty to make it happen in a practical transfiguration of the everyday.

To the eye of reason, I repeat it, it certainly seems strange. But then the majority of human actions are not meant to be looked at with the eyes of reason. (A. Huxley 1948, 47)

‘The impulse of research must proceed not from philosophies but from things and from the problems.’ In this way, Husserl (1965, 146) famously presented his basic conviction concerning the idea of a phenomenological philosophy. In what follows, I attempt to relate this conviction to the momentous challenge that one of Husserl’s major successors, Merleau-Ponty, has written into the diary of the ‘phenomenological movement’. In a widely known reflection Merleau-Ponty not only held that the philosopher carries his bodily shadow with himself, a shadow that, as he put it, is ‘not simply the factual absence of future light’ (Merleau-Ponty 1964, 178). Still more radically, he also contended that we need to include *non*-philosophy into philosophy – and to understand its relationship to its other.^1^ This move indeed appears of paramount importance to me, yet I propose to pursue the confrontation of philosophy with its other into a strikingly different direction than Merleau-Ponty did. In a nutshell, I intend to focus on those forms of otherness that violence, religion, and especially ‘religious violence’ pose for a type of philosophy that understands itself as a continuing reflection upon reason, its assumed teleology, and its intrinsic crises.^2^

In the background figures my conviction that phenomenology, given its critical attitude towards modernity, offers us the appropriate means to unreservedly confront these vexed phenomena, that is, without reducing them to the surreptitiously claimed measure of either *one* reason or one *true* religion (be it a religion of reason or of the spirit). This claim for a truly phenomenological confrontation with these phenomena testifies to a high hope. I wager that phenomenology is capable to confront violence and religion without representing these phenomena as ‘the other of reason’. Indeed, the unanimity of occidental reason easily tends to indifferently encase itself by way of excluding (or indeed deporting) these phenomena from the bonds of reason alone. Such a move, finally, only results in rendering them even more monstrous and in hiding the self-righteousness of reason as such, equipping it with a ‘good conscience’. Given this, the philosophical dispute over religion and violence can only take place via a confrontation with the most basic questions of philosophy as such.^3^ To my understanding, phenomenology offers an adequate approach to provide a concrete account of these phenomena and the basic problems they entail.

In what follows, I wish to demonstrate why this is the case and how such an analysis can proceed. In a first part, I will consider briefly the current debate on the post-secular condition and wish to demonstrate why the questions concerning the so-called ‘return of religion’ and ‘religious violence’ appear to be intrinsically interlinked. I hypothesize that we need to analyze religious violence in the ‘post-secular context’ in terms of an ‘originary supplement’ of ‘disengaged reason’ rather than in terms of mere chaos, senselessness, atavism, or monstrosity – terms which, as we witness today on an immense scale, are strongly endorsed by the contemporary theater of cruelty committed in the name of religion(s). (1) In a next step, I argue that in order to confront the specificity of ‘religious violence’ beyond traditional (i.e. instrumental, responsive, habitual) explanations, a novel phenomenological account is needed. Here I critically examine the potential of contemporary phenomenologies of religion and opt for developing an integrated phenomenological account, i.e. one that enables us to trace the phenomenality of religion in terms of a correlation between the originary givenness of transcendence and capable man’s creative capacities to respond to it. (2) Following Ricœur, I finally discuss ‘religious violence’ in terms of a monopolizing appropriation of the originary source of givenness. As I wish to demonstrate, this appropriation entails the conflation of man’s finite freedom to poetically respond to the transcending appeal of the foundational with the surreptitiously obtained sovereignty to *perform* it in practices of self-transcendence that transfigure everydayness and the many disavowed violences it entails.

## 1. The context of post-secularism: the ‘return of religion’ and the problem of ‘religious violence’

Key for what follows is my claim that philosophy, if it is to be understood as a theoretical reflection on the foundations of human reason and its practical implementation has to realize itself in the course of a self-reflective clarification of its relationship to its other(s). A prominent guiding thread for this venture is the problem of *violence.* Violence, however, has all too frequently been eclipsed, suppressed, or overdetermined in the vast majority of classic and modern positions in political and social philosophy. Without a doubt, this does not amount to saying that violence has been absent from this discourse. It rather visibly appeared on significant points in the occidental philosophical discourse as the true medium of the question concerning the ‘human condition’. In this context, one might simply refer to the topic of *war* which has provided our whole philosophical tradition – from Heraclitus and Plato, via Hobbes and Kant and German idealism to contemporary thought – with perhaps the most important exemplification of this foundational problem. And more generally viewed, political modernity has definitely defined itself by its attempts to ban or at least enclose war and violence in the name of man-made rational order. The problem however is that this undoubtedly laudable project also remains inauthentic toward the violence it sought to ban by transforming it into legitimate manifestations of power, i.e. a kind of fuel for our modern social technologies of order. The respective philosophical discourse since Hobbes that emphasized the human producibility or facticity of such order also lapsed into a sort of performative blindness for the essential contingency of such order. Without a doubt, the meaning of human practice became definitely uncoupled from the mythical idea of a basic cosmological order in this era. The subsequent inauguration of a teleological order, however, generated its own set of problems: first and foremost, it crystallized in the semantics of the ‘process of civilization’ and related concepts of progress. This semantics is still prevalent and catchy today, especially in the context of globalization, but basically results in nothing but freezing the ‘normativity of the factual’. That modernity in its unanimous orientation towards order might indeed have given birth, to summarize Zygmunt Bauman’s (1991, 58–59) account, to a ‘monster’, expresses an important insight in this regard: it points to the fact that the very attempt to contain violence in the fabrics of social order by subjugating it to the claim of legitimization, has at once eclipsed the historic genesis of the binary code of legitimacy/illegitimacy and has thus obnubilated the way it is itself rooted within a variety of disavowed societal violences (Farmer 2004; Kleinman 2000; Whitehead 2007). Violence, thus viewed, has been rendered ‘the illegitimate per se’ and order its unanimous other. That, however, this code revolves around a ‘societal pre-structuring of thought’ (Luhmann 1974, 228), which lacks sensibility for a reason ‘in the making’, refers to the sore point. This point is clearly epitomized in the traditional separation of reason and violence, which paints an all too flawless picture of the ‘order of things’ (Waldenfels 1991). By adopting figures of thought like, e.g. the ‘bond of separation’, ‘identity in difference’, or the ‘unforced force of the better argument’ as Habermas (1996, 306) put it in terms of social theory, our dear tradition of thought follows the idea of a unity of reason that pretends to achieve consensus in understanding. Such a move, however, as Levinas has insistently objected, in fact re-establishes the reign of but one theory or, still worse, the violent primacy of totality that has so deeply shaped occidental philosophy: ‘Coherent discourse is one. A universal thought dispenses with communication. A reason cannot be other for a reason’ (Levinas 1979, 72).

Levinas’ discussion of the idea of totality that has preoccupied philosophy since Parmenides, focuses the violent exclusion of the otherness of the other that the thought of totality entails. In this context Levinas confronts a variety of exemplary philosophical assessments. His focus is not only on Husserl’s transcendental subjectivism, which locates the absolute in man. He also criticizes Heidegger’s ‘fundamental ontology’, which results, to cite Patočka, in the warlike ‘irrationalism of a pre-existent Being lacking all human closure, all practical value’ – a process which thus ‘leaves entirely aside what man is and can be to man’ (Patočka 1989, 271). Finally, another and indeed basic target of Levinas’ critique is, as far as I see it, undoubtedly Hegel. Hegel’s dialectics – according to Sartre ‘the very image of violence’ – is not of incidental importance to Levinas (Sartre 1992, 184). As an attentive reading proves, Levinas’ thought revolves around an implicit but nuanced discussion of Hegel’s dialectical philosophy of totality and especially its implications for social philosophy. Following Balbontin-Gallo (2015), I hypothesize that a major leading clue of Levinas’ critical discussion of Hegel can indeed be found in the ‘operative concept’ of the so-called ‘struggle for recognition’. As is widely known, Levinas’ attempt to confront the excessive and unassimilable alterity of the other who escapes totality hinges in principle upon a claim that is said to arise from *beyond* the normative framework of reciprocal recognition. Viewed against this background, Hegel’s dialectical account of recognition seems to epitomize the thought of totality par excellence for Levinas. Yet, in the context of Levinas’ attempt to come to terms with the irreducible practical necessity to mediate one’s absolute ethical responsibility for the other in the framework of justice and politics, the figure of the ‘third party’ (*le tiers*) undoubtedly testifies to the unmitigated presence and importance of this question. I am, however, not at all concerned here about the intrinsic coherence and the validity of Levinas’ attempt to provide an adequate interpretation of the intersections between the ethical and the political (Bernasconi 1999) in terms of the field wherein the recognition of ‘the other’ might take place. Rather, I want to stress the fact that Levinas’ challenging attempt to think against the totalizing tendencies of occidental philosophy becomes problematic in itself once it confronts the very ‘struggle for recognition’ that is led by *others* – a struggle that for him appears to betray one’s very otherness for the sake of collectively shared differences. To this testifies the immense problematic that Levinas’ finds himself engulfed in face of a truly *different* other, i.e. one who does not share the assumedly universal and, hence, invisiblized cultural heritage that is taken to provide the proper space for the recovery of reciprocal relationships of recognition: to be more precise, I have in mind the *religious* other and his practices – practices whose meaning does not work out in the intelligibility of the heritage that conjoins Athens and Jerusalem. On the one hand, confronted with the question ‘Who is my neighbor?’ (and who *not*!) – where the notion of the ‘neighbor’ apparently denotes the paradigmatic figure of the other – Levinas’ (1979, 299)response appears quite unambiguous: ‘the subject is a host.’ Yet, on the other hand, there also appear others in Levinas’ discourse who are simply said to be *wrong* and, thus, prove unworthy of the very otherness they embody: if ‘all the rest’ is indeed apostrophized as ‘dance’ and ‘must be translated’^4^ – then all this should make us wary in the context of a position that ponders about hyperbolic responsibility, the un-condition of being hostage for the other, traumatic substitution, and so forth. And indeed, this problematic is reflected most clearly in Levinas’ incapacity to adequately confront and respond to the claims of the religious other, her kind of lived difference that always already declines her otherness, and hence makes her vulnerable not only in her absolute alterity but also in her specific difference (Moyaert 2008).

In order to connect this reflection with the preceding line of thought, we may conclude that even Levinas’ philosophy which most radically seeks ‘the inclusion of the other’, remains inauthentic vis-à-vis its own violence(s). Exactly this danger gains further acuity in regard to the recent debate concerning the ‘post-secular society’ where this inauthenticity further leads to the suppression of the said violence and perhaps even to its projection onto its others. This debate demonstrates exemplarily how the misperception of the religious other in terms of irrationality, atavism and ‘violence incarnate’ feeds into the construction of a ‘myth of senseless violence’ (Blok 2000; Whitehead 2007) that finally serves to justify the *ultima ratio* of the counter-violence that our ‘culture of conflict’ does not call by its true name. Thus viewed, this myth leads to a structural eclipse of the normatively embellished violence of our universalized political claims in the name of their ethically disguised unconditionality.

The *extraordinary* and, hence, implicitly irrational, violent, etc., status attributed to religion provides an exemplary case of this tendency. Lately, this problematic surfaced very clearly in Habermas’ attempt to re-define the role of resurgent religion in the framework of his conception of discursivized reason. To cut a long story short, even in this sophisticated design religion in the last analysis retains the nimbus of the disorderly, i.e. the opaque, consequently *à la limite* irrational, and (at least potentially) violent. We realize this in painful but perfect clarity in one among Habermas’ most recent reflections on religion. In being confronted with the apparently unconditional claims of reason’s other, we have, argues Habermas, in fact only one way to react: we may only ‘let ourselves be instructed by the arguments of others about the blind spots of our own self-understanding regarding our principles and their practical application on the basis of a self-conscious defense of universalist claims’ (Habermas 2013, 293).^5^ Everything else would result – at least on my understanding of Habermas – in a sort of performative self-misunderstanding of reason (an interpretation that mirrors Kant’s attempt to found the ‘categorial imperative’ in the *Groundwork for the Metaphysics of Morals*). If we, however, unmask the rather euphemistically so-called ‘arguments of others’ as a cipher for the extraordinary claims of the other, which are only referenced *belatedly* in order to strengthen the sovereignty of reason’s own (and selfsame) identity (Derrida 2005; Yegenoglu 2014), we see clearly what is at stake: in conflating the transfiguring power of the extraordinary with the indeed extraordinary powers of imagining disorder, such a gesture is but handing a free pass for *discursivized* reason to perceive in the other’s extraordinariness nothing but its opacity to the eye of reason and, hence, an intrinsic threat of disorder (or chaos). In one and the same gesture, however, the projective construction of the extraordinary other in terms of a disordering potential is eclipsed and disavowed. In other words: it might indeed be the case that we (yet) understand ourselves wrongly, that we (yet) apply our allegedly universal principles inadequately, yes, all this might be the case. The fact, however, that the pre-given (orderly) location of the ‘we’ that speaks so and the (normative) principles derived therefrom might themselves be founded upon presupposed conditions like, e.g. the eclipse of the ‘moral emotions’ from the genesis of modern rationality (Steinbock 2014); that their unconditional claim for universality might itself be surreptitiously held up and disavow the violences it entails, all this remains totally unquestioned within the spell cast by this inherently Western conception of an ‘ordering reason’ that got used to confronting again and again its ‘raw material’ to proceed further on (Bauman 1989).

Given this, we need to ask if the appeal of the other to be perceived, respected, and recognized as an other is not sacrificed here for the claim to rescue a ‘true’ other (Abu-Lughod 2002); we might also ask if the normativist claim to ‘contain and translate religious identity’ is not realized here ‘at the expense of intrinsic alterity’ (Kearney 2011, 106). Such a move, however, is not at all innocent. It rather amounts to a sacrifice that at once eclipses the self-righteousness of an intrinsically totalizing and ‘indifferent reason’ (Liebsch 1999). And still worse: in the last analysis this very tendency even seems to provide an absolution for a practice of reason that closes itself off in an auto-immunizing way. Thus viewed, however, it finally seals the ‘conflation of freedom and sovereignty’ which has been exposed by Arendt (1998, 254–255) as the original sin of modern political theory: in this respect, reason finally translates itself into an unconditional politics of ‘rational assimilation’ (Kearney 2011, 102–104) that leaves no room for ambivalence and an ‘untranslatable remainder’ (Bergdahl 2009)^6^ but demands that its claims are to be met without condition if one wants to count at all as a *reasonable* being.^7^

This perhaps all too quick discussion still mirrors very well the current situation, its disarray or crisis, which call upon us to rethink our tools of coming to terms with the vexed realities that the ‘return of religion’ entails. It is, of course, beyond any doubt that ‘unconditional claims’, whoever raises them, create regimes of perception, interpretation, and action that are prone to violence (Liebsch and Staudigl 2014). ‘Crudely put, one cannot compromise over the holy without compromising the holy.’ Margalit’s (2010, 24) famous formulae renders the basic problem for the ‘religious mindset’ very palpable. But it is also a fact that ‘secularized reason’, for its part, got used to legitimize violence qua justified counter-violence in order to preserve itself for the sake of upholding the universalist aspirations it proclaims to serve. And indeed, even the most rigorous (democratic) claim to keep the center an ‘empty place’ (Lefort 1988, 232–233), that is, to not fully symbolize the essence of community in whatsoever terms, necessarily verges towards the unconditionality that it otherwise refutes: isn’t the very claim that refutes absolute truth claims (e.g. concerning a revealed religious truth) itself an absolute claim that possibly justifies violence in order to preserve (reason) itself?

While it appears to be beyond doubt that (at least theistic) religions with their generic ‘promise of salvation’ (Riesebrodt 2007) – be it in terms of retaining an unscathed community or of providing means to manage human finitude and contingency – embody a (perhaps *the*) *truly unconditional claim* par excellence, it is also high time to acknowledge various auto-idolatries of occidental reason that have rendered its assumedly liberating project inherently doubtful. This is not only true of the Manichean implications of the so-called ‘war on terror’ after 9/11 but also of more considerate attempts like the unwillingly hypocritical usage of the universalist category of ‘world cultural heritage’ in the earlier stages of the conflict with the Taliban concerning the Buddha statues in Bamiyan (Staudigl 2014). In another context, we also need to think about the economic hubris or rather self-abandonment of reason to the idolatrous forces of the market which creates a manifold of disavowed violences in the maelstrom of globalization (Appadurai 2006, 35–48): given that human finitude is being more and more related to the existential fact of the exhaustibility of natural resources, one need not wonder that religious semantics are becoming increasingly present in this regard, too. Finally, the related ‘new wars’ that disseminate today – wars in which warring parties seem to gain more from waging war than from making peace – are not only due to the global asymmetry of power relations and a far-reaching economization of war that transforms whole precarious classes into ‘cultures of violence’. They also attest to the semantic potentials of religious systems of knowledge to justify violence on the part of those who experience themselves being outcast from the very symbolic space where participation in a liberating ‘struggle for recognition’ is only assured. In all these contexts, thus viewed, major patterns for the justification of religious violence can easily be brought to the fore (Clarke 2014). I argue, however, that it is all too easy to reduce the philosophical question of religious violence to a mere matter of the discursive legitimization (or delegitimization) of such violence. What is dangerously excluded or misperceived in such a perspective, are (once again) our embodiments (Mensch 2009) and the role of the ‘moral emotions’ in our contemporary ‘social imaginaries’ (Steinbock 2014). In a nutshell, eclipsed is what Ricœur (1986) has termed the ‘affective fragility of man’ and the various ways they feed into what may be called the *affective teleology of violence*.

What this means can be clarified with regard to a widespread explication of violence in terms of an instrumental response to a denial of recognition, an explanation which is quite prevalent in current social and political theory. Instrumental rationality may indeed be of major relevance in such cases, but still more basic is the fact that denials of recognition, be it in form of bodily violation, legal degradation, or cultural humiliation are not simply perceived on a merely cognitive or discursive basis. They are rather experienced in flesh in a variety of negative affects like shame and anger (Honneth 1995, 121, 135–139, 164). As Honneth (1995, 164) exemplarily holds,

social shame is a moral emotion that expresses the diminished self-respect typically accompanying the passive endurance of humiliation and degradation. If such inhibitions on action are overcome through involvement in collective resistance, individuals uncover a form of expression with which they can indirectly convince themselves of their moral or social worth.

This argumentation clearly testifies to the relevance not only of human affectivity but also of ‘affective economies’ and even a ‘cultural politics of emotions’ (Ahmed 2004a, 2004b) in the etiology of performative, i.e. empowering ‘counter-violence’. Exactly this still largely underexposed intersection of social action and affectivity, of ‘social sense’ and ‘the somatic’, is the point where phenomenology has to come in: it is here that it can help us to provide a deeper understanding of what is at stake in the phenomenon we are dealing with – the irreducible intertwining of reason and affectivity that makes ‘religious violence’ an inherently ambiguous yet also exemplary phenomenon. Only seemingly paradoxically, the rational instrumentality of violence here goes hand in hand with an auto-telic function that radically transfigures the ordinary with its excessive ‘phenomenal’ display (de Vries 2013).

To sum this up: in the contemporary ‘crisis of immanence’ (Höhn 1996; Taylor 2007) where the promise of emancipation generates increasing solitude and the blessings of technology and autonomy induce overstraining challenges for the individual, religion is far from disappearing – quite the contrary. In contradistinction to the longstanding narrative of secularization that preached religion’s future societal insignificance, its unbroken competence to generate social meaning now seems to receive an ever higher rating when faced with late modernity and its discontents. Not only the creation of new spiritual imaginaries (Knoblauch 2009) and the vibrant comeback of ‘political theologies’ (De Vries and Sullivan 2006) but first and foremost the upsurge of unprecedented forms of religious violence attests to an unforeseen and inherently ambiguous afterlife of religion in a time that was expected to do away with it. Especially the community-instituting potential of religion, which has culpably been overlooked in the vast majority of recent discourse, mightily comes to the fore again in this context (Braeckman 2009). The empowering ‘liturgies’ of religious violence, as Kippenberg (2013) respectively argues, concretely embody this potential in a most specific and intriguing way.

At this point the question *why* to provide a *phenomenological* account of such violence now lays clearly at hand: we indeed are in deep need of such an account that avoids falling prey to the ‘fallacy of legitimization’, that is, to portray such events either in terms of instrumental action, as a legitimate response to some preceding violation or threat, as a habitual disposition (as recently widespread talk about so-called ‘cultures of violence’ insinuates), or to otherwise simply relegate it to the realm of the senseless (Kippenberg 2011a). The question yet remains *how* such an account is to be shaped. It is exactly this question that I attempt to confront in the remainder of this paper. In this context, the fact that the so-called ‘return of religion’ involves its *transformation* (Derrida 2002) and *dispersion* into secular life-worlds (Höhn 2007, 33–50), is not a contingent and negligible factor but rather of paramount importance. It is indeed at this juncture where we touch upon the central phenomenon that should concern us – the seemingly paradoxical intertwining of reason and its technological avatars with its assumed other, i.e. religion (Derrida 2002). Thus viewed, I propose to focus religion and religious violence in terms of the *intertwining of experiences of transcendence and practices of self*-*transcendence* that they appear to revolve around. To confront this intertwining, an integrated phenomenological account is needed. In the following section, I will explore the potentials of contemporary phenomenology to develop such an account.

## 2. Two current paradigms in phenomenology of religion

After a longer period of structural disinterest, French phenomenology has demonstrated strong interest in religion more recently. Yet the proponents of the so-called ‘theological turn’ in French phenomenology have also been criticized on many occasions for rejecting the ‘methodological atheism’ of phenomenology and for turning it into a pathfinder for a theology in disguise. For their recourse to concepts like the ‘wholly other’ (Levinas), the ‘immanence of absolute Life’ (Henry), or ‘the saturated phenomenon’ (Marion) these ‘new theologians’ have been reproached for violating the methodological rigor of phenomenology and for a ‘theological hostage-taking’ of phenomenology (Janicaud 2000). In light of this polemic, the ‘theological turn’ has been received only marginally and rather unproductively until very recently. I argue, however, that exactly this polemic understanding has kept us from assessing the vast potential phenomenology harbors for developing a critical philosophy of religion.

As Kosky (2001, xix) has shown for the case of Levinas, the analysis of core concepts developed in contemporary phenomenology (in this case *responsibility*) ‘opens onto a philosophical articulation of religious notions and thus makes possible something like a philosophy of religion’. I argue that the same can be demonstrated for Henry, Marion, Chrétien, Ricœur and others, who, according to my hypothesis, all provide interpretations of our finite but creative capacity to respond to figures of ‘unconditional givenness’: the *unassimilable gift of the other* in Levinas, the inherently *exceeding gift character of all reality* according to Marion (2002a, 2002b), the intuition of *Life as a gift* ‘which cannot be refused’ in Henry (1973, 475; 2003), the *gift of a call to be responded* in the ritual offering of a world in Chrétien (2004), or the ‘logic of superabundance’ and the ‘*economy of the gift*’ that it entails in Ricœur (1995, 315–329) – all these phenomena clearly attest to ‘religious meanings that have traditionally been consigned to the unintelligibility of faith or else reduced to the intentions (conscious or unconscious) of the self’ (Kosky 2001, xix). Instead of serving a veiled theological purpose, a phenomenological *reassessment* of these figures of givenness will serve as a relief for philosophy of religion (and perhaps also for theological thought).^8^ As to my understanding, it does so since it keeps philosophy from ‘sacrificing the significance of religious notions at the threshold of intelligibility and understanding’ (ibid.) and, thus, helps it to overcome the extremes of both the assumed autonomy of reason as well as the heteronomy frequently affiliated with faith.

As the afore-mentioned list demonstrates, the ‘semantics of the gift’ and a related reflection on its un-economic superabundance runs like a red thread through contemporary French phenomenology. Despite the obvious differences between thinkers like Levinas, Henry, Derrida, Marion, Chrétien and Ricœur, their analyses still appear to revolve around one shared cardinal insight. This consists not simply in addressing, like Husserl early on proposed, the manifold ‘*how* of givenness’ but rather in acknowledging the bare facticity (*aseity*) of givenness as well as the finite freedom of the one who is called to respond to its summons. This general insight definitely is dealt with very differently in these accounts, being related to different interpretations of who comes ‘after the subject’, to varying assessments of a ‘topics of givenness’, and especially to diverging interpretations of the nature and scope of the response to the call of the given. For reasons of space, I will restrict my discussion here mainly on Marion and Ricœur’s exemplary, divergent but also reciprocally enriching accounts. A critical synthesis of some among their major insights shall help me to pave the path for the integrated account I have in mind.

In his systematic outline of a ‘phenomenology of givenness’, Marion (2002b) has demonstrated that the focus on givenness renders phenomenology sensitive to the genuine yet inconspicuous experiencability of the phenomena in their very possibility beyond the requirements of being actually given. Pursuing the question ‘How must one proceed phenomenologically in order to return to the “things themselves” of religion?’, he accordingly searches for a phenomenological account to assure the phenomenological status of revelatory phenomena, i.e. the ‘general possibility of revelation’ (Marion 2008, 1–17). The related ‘broadening of phenomenality’ that this affords us is presented in terms of their *unconditional givenness*: the autochthonous ‘élan of givenness’ unfolds, as he holds, beyond the classical phenomenological presuppositions of (a) a constituting Ego/I and (b) a signifying horizon (however it may be designated: in terms objectivity, Being, flesh, ethics, etc.). In suspending the axiomatic character of these two presuppositions that originally converge in the concept of intentionality, Marion attempts to re-establish the *primacy of givenness* and to bestow a phenomenological status to so-called ‘impossible’ and ‘unconditional phenomena’, or ‘paradoxa’. His analyses of ‘saturated phenomena’, i.e. phenomena that revolve around an ‘excess’ of intuitive givenness over the intentional meanings worked out by the ego, testify to this primacy (Marion 2002a, 2002b). They furthermore unfold it in a variety of thematic regards (historic event, work of art, embodiment, otherness), including, in the last analysis, also the religious phenomena of revelation. As to his understanding, the latter are distinguished specifically by the fact that they embody a higher, ‘second degree’ of saturation (Marion 2002b, 235) which involves the collapse of all the categories of thought as the traditional means to conceptually think what is given. In distinction from the other types of saturated phenomena, the ‘doubling of saturation’ in revelation implies not only a reversal of the gaze, a multiplication of sedimented horizons of meaning, and a disqualification of intentionality that affects the subject in its innermost self and constituting power; it ‘rather demands *devotion* from the subject’, thus transforming it truly into the ‘gifted’ (*l’adonné*). Put differently, the phenomena of revelation ‘raise the traits of the saturated phenomena into boundless heights’ (Gondek and Tengelyi 2011, 190).

Without delving deeper into Marion’s rich and well-commented studies of various ‘saturated phenomena’ here (Marion 2002a; cf. Gschwandtner 2014), it has frequently been criticized that his ‘deduction’ of a possible *phenomenology of religion* from the ‘objects of religion’ (Marion 2008, 1–2) does not involve the same methodological neutrality as his other studies of ‘saturated phenomena’ do (Janicaud 2000, 50–69). Accordingly, he has been reproached on manifold occasions for privileging a specific set of religious manifestations, especially ‘the manifestation of Christ […] as [a] paradigm of revelation’ (Marion 2002b, 236), in order to factually deduce the ‘pure possibility’ of revelation.^9^ To assess the merits of his account suchlike, however, is misleading. Indeed the question whether or not his work may sometimes transgress the border between phenomenology and theology is not of paramount importance here. This sort of criticism rather all too quickly tends to lose sight of the truly important insight that his change of perspective regarding the phenomenality of unconditional givenness entails. After his earlier theological writings, which indeed provided a phenomenological underpinning for posing theological questions in a post-metaphysical framework, Marion’s more recent attempt to elaborate on a phenomenology of givenness proceeds differently: as to my understanding it proposes nothing but to trace experiences of unconditional givenness in general (and of a transcendent god, epitomized in the superabundance of love that reaches ‘beyond being’, in particular) within the confines of immanence alone. In this truly phenomenological perspective unconditional givenness, finally, does not only become an issue in a recourse to theophanies or epiphanies anymore. It is rather revealed as a generic *donative* (*dativisch*) dimension of the phenomena that Marion (2008, 119–144) explores also explicitly with regard to their everydayness or ‘banality’.

This move, however, testifies not only to the general *irreducibility* of givenness*.* In its basic transcendence this dimension is also resistant, as he frequently emphasizes, to mere categorical analysis and conceptual thought. Accordingly, the ‘primacy of givenness’ bears, to speak with Schelling, a truly *immemorial* (*unvordenklich*) character. As Schelling’s related critique of ‘negative philosophies’ has demonstrated convincingly, any attempt to assess it by means of thought and its conceptual devices is doomed to fail. Following Ricœur (1999, 3; 2010, 35) and Zaborowski (2004), I hence argue that we need to take up phenomenology in the light of Schelling’s respective idea of a ‘positive’ or ‘aposterioric philosophy’ (Schelling 1979, 211–212; cf. Marion 2002b, 18)^10^ in order to open up a productive venue for a *post*-*foundational phenomenology of religion* that lets itself not be overdetermined by metaphysical interdicts or theological presuppositions. And as far as I see, Marion’s account indeed offers us an important missing link for such an undertaking. In conceiving givenness in terms of saturation, i.e. in inherently *affective* terms, it demonstrates two things: first, it exposes phenomenology to what *befalls* experience^11^ and thus frees it from the metaphysical yoke of ‘sufficient reason’ (Marion 2008, 5); second, this exposure also radically transforms phenomenology as such alongside a novel correlation that overthrows or delimits the representational sway of intentionality: the correlation of *appeal and response* (Gondek and Tengelyi 2011, 190–206; Marion 2002b, 282–296). According to this correlation, givenness is recovered in the experience of something extra-ordinary that calls upon the subject to respond to its summons. Put differently, confronted with an excess of intuition over the intentions worked out by the ego, our preconceived conceptual grip on the world is challenged and the instituted meanings it entails are shaken. In responding to the ‘immemorial’ (Marion 2002b, 295) call of saturated givenness, its summons is articulated in meaningful yet always immature form. Givenness, thus viewed, in the last analysis appears to invest all phenomena with a ‘pure form of the call’ that attests to a ‘nameless voice’ (Marion 2002b, 296–308).

Given this the idea of a ‘phenomenology of religion’ rests completely on the premise of such ‘unconditioned phenomena’. Their excessive constitution is turned into a pattern of phenomenality as such, investing it with a gift-like, i.e. inherently un-economic nature. On the one hand, this attests clearly to the eminent possibility to rethink God in terms of the gift (Horner 2001). On the other hand, however, it manages to do so, as Waldenfels (2012, 118–119, 406–407) objects, only on the basis of a surreptitiously claimed direct access to the invisible. Be it the ‘pure form of the call’ and the ‘fold of givenness’ in Marion or an ‘essence of manifestation’ that Henry (1973, 2003) locates in ‘auto-affective life’, this reader argues that in both cases the foundational concepts of ‘pure givennness’ or ‘auto-affection’ are read solely in terms of their assumed auto-intelligibility but not in terms of the ‘significative difference’ that we need to disclose them in flesh.^12^

In fact, both Marion and Henry do not completely disregard the ‘finitude of phenomenality in the realm of givenness’ (Marion 2002b, 310). What the former calls ‘abandoned phenomena’ and what Henry (2003, 133–151) discusses in terms of ‘transcendental forgetfulness’ rather carves out some space for the hermeneutic or narrative articulation of the ‘radical immanence’ wherein the ‘play of the call and the responsal’ (Marion 2002b, 308) are said to take place. Marion’s later attempt to excavate an inherently hermeneutic dimension of givenness (Marion 2013; cf. Marion 2002b, 308) as well as Henry’s (2012) latest attempt at understanding the irreducibly duplicitous nature of language clearly attest to this fact. Thus viewed, Waldenfels’ rigorous criticism that the focus on the absolute self-givenness (be it of ‘unconditioned phenomena’ or the auto-revelation of ‘Life’) totally eclipses the alterity of the given has to be softened at least a bit.

Yet another problem is of paramount importance for a phenomenology of religion, namely *a limited understanding of embodiment*. At a first glance this kind of criticism might appear counterintuitive. Doesn’t Marion (2002a, 82–103, 2002b, 231–232) count the flesh among the ‘saturated phenomena’ and hasn’t Henry been preoccupied with the ‘phenomenology of the body’ from his inaugural attempt to unthink the ‘essence of manifestation’ until his latest works (Henry 1975, 2015)? All this is definitely true. Yet it still seems to me that the focus on saturation and auto-affection leaves largely underdeveloped the ‘strangely intimate exteriority of flesh’, Marion (2002a, 103) mentions only in passing. To be more precise, it eclipses, as Falque (2004) and Waldenfels (2012, 118) also see, the various facets of a bodily self-withdrawal of the incarnate subject and, hand in hand with this, its inherently intersubjective or rather inter-corporeal constitution.^13^

In both cases, a true ‘phenomenology of the inapparent’ (Heidegger 2003, 89), which would revolve around the interplay between instances of hyperbolic givenness and their irreducible withdrawal in our responses, is not to be found. For this is substituted a ‘phenomenology of the invisible’ which is not ‘of this world’ (Merleau-Ponty 1968, 151) but rather seems to live from mere ‘incantations’ (Janicaud 2000, 27, cf. 86) to the absolute. Thus viewed, both Marion and Henry’s thinking of givenness or self-giving does not really pave a path for a genuine phenomenology of religion. Both rather deal with figures of an ‘immemorial gift’ that reverberates in the nocturnal depths of the soul or the ‘immanence of givennness’ as such. Thus viewed, these thinkers definitely open rich pathways for a phenomenological theology (Rivera 2015) but definitely not for a phenomenology of *religion*. This is not achieved since the correlation between call and response remains too formal and underdeveloped in the case of Marion, where the ‘gifted’ (*l’adonné*) ‘receives itself entirely from what it receives’ (Marion 2002b, 268).^14^ It is also not achieved in Henry’s work, where finally nothing but the ‘original drama of life that is God’s presence inside me’ (Rivera 2015, 328) is recovered in this correlation, thus leaving no space for either human creativity or even an eschatological horizon. Without a doubt, concepts like ‘barbarism’ or ‘abandon’ are nominated by these thinkers as possible denegations of the gift’s creative appeal but in the last analysis such negativity is fully recovered by a ‘second birth’ (Henry 2003, 152–170) or transformed into an unbreakable ‘obsession’ for ‘the night of the unseen’ (Marion 2002b, 319).

Viewed against the backdrop of this criticism,^15^ let me turn now to Ricœur’s account of *religion.* As we will see soon, the correlation of call and response is also at the center of his account. But before we comment on his way of unfolding this correlation, we need to note first that the methodological status of religion in Ricœur’s work is also far from being undisputed. In this regard, it has frequently been mentioned that he did not include the more religiously orientated ‘Gifford Lectures’ into his opus magnum, *Oneself as Another*. And indeed, on the one hand, he himself asserted on manifold occasions that given his firm commitment to biblical faith he wanted to maintain an avowed ‘methodological atheism’ in his philosophical works. Given his own explications, this seems to be the case in this later book, too, which eagerly seeks to avoid any ‘cryptotheology’ (Ricœur 1992, 24). His ‘biblical hermeneutics’ (Ricœur 1980, 1995) and earlier studies in religious narrative and symbolism (Ricœur 1969), on the other hand, definitely draw from these sources. They, however, explicitly did so with a clear and outspoken philosophical motivation: Ricœur indeed nowhere raised an argument for substituting a ‘cryptophilosophical framework’ that could be found in the biblical texts for the loss of ultimate foundations that the ‘shattered cogito’ implied. Such ‘controlled schizophrenia’, however, sometimes proved difficult to handle. And especially in his last works on recognition and forgiveness it becomes very clear that he borrows concepts from both fields as, e.g. the role assigned to the *agape* in the discourse on recognition clearly shows (Gschwandtner 2012, 10).

Yet the vexed question concerning the relationship between philosophy and theology and its methodological reflection is not the important issue here. Systematically viewed, I rather argue that a ‘phenomenology of religion’ revolving around the correlation between the immemorial gift of a call and the creativity of human responses, is something that could find an adequate placing in the account of *Oneself as Another*, even if the author has expressly excluded it (Ricœur 1992, 23–25). If this book’s hypothesis concerns the fact that the easiest – and perhaps only possible – way to the self is via *the other*, doesn’t its systematic unfolding indeed even require that religion be included as an *exemplary form* of dealing with such otherness? Whereas Ricœur (1992, 215) mentions it in his chapter on ‘The Self and the Moral Norm’ in the context of an analysis of evil as something that radically ‘*affects* the use of freedom’, it is nearly completely absent from the last chapter of the book. This is all the more astonishing since this is the place where Ricœur attempted to explicitly found his ‘new ontology’ on the difficult relationship of ‘selfhood and otherness’, exploring it in terms of an interplay between irreducible ‘experiences of passivity’ and ‘multiple ways’ of responsive ‘human action’ (1992, 318). Thus viewed, the basic correlation between affective call and creative response becomes graspable in its most comprehensive scope and overall importance. Ricœur here not only attests to a yet unfathomed *plurality of otherness* but also provides us with an idea of how his never explicitly elaborated idea of a ‘poetics of the will’ (1966, 26–33, 191–192, 415, 425) might look.^16^ It is indeed in this late context, by taking up his conception of ‘narrative identity’ and re-calibrating it in regard to the relationship of the voluntary and the involuntary (which grounded his thought from the very beginning onwards: Ricœur 1966), that this idea resurfaces. In other words: with the emphasis being put on the correlation of call/response, the conception of ‘narrative identity’ now is taken to refer most generally to the self’s ongoing and dynamic interpretation and volitional ‘making’ of its own meaning in a world that confronts it with manifold otherness. It is, as Wall (2005, 35) puts it, ‘the final site within which the self may respond to its own inner disproportion of finitude and freedom’.

Since the self in its confrontation with otherness constitutes itself in a dialectic interplay between its given *identity* and functioning *ipseity* (1992, 1–18)*,* it has never a ‘stable and seamless identity’ but rather bears the capacity ‘to weave different, even opposed, plots about our lives’ (Ricœur 1985, 248). Narrative identity basically revolves around an element of *free self*-*creativity* that Ricœur proposes to understand in analogy to the reader’s capability and responsibility of transforming texts into personal and collective meaning. The link of this conception to the idea of a ‘poetics of the will’ is explained clearly by Wall (2005, 36) again, who states that

this making and unmaking of oneself is reminiscent of Greek *poiêsis* as making, forming, crafting, creating – but now applied to the self’s own very person. The self understands and invents itself ‘both as a reader and the writer of its own life’.

Interestingly, it is with reference to the correlation of call/response that Ricœur takes up the problematic of a ‘phenomenology of religion’ in the chapters he did not choose to include into *Oneself as another*. As to his understanding, as we can already find it expressed in his early work, a ‘phenomenology of religion’ must not start with a direct philosophical appropriation of transcendence in the classical sense.^17^ It rather has to start from and revolve around the vast variety of religious ‘feelings and dispositions’ that ‘transgress the sway of representation and, in this sense, mark the subject’s being overthrown from its ascendancy in the realm of meaning’ (2000, 127). These ‘absolute feelings’ embody ‘ways of being absolutely affected’ which serve as ‘test cases that bear witness to phenomenology’s inability to open the intentionality of consciousness to something completely other’. To this ‘Other who affects’ from beyond intentionality correspond, as he adds, responsive ‘dispositions that can be placed under the general heading “prayer”’ (2000, 128) but in fact include a vast variety of dispositions (like, e.g. complaint, praise, supplication, demand, etc.). Ricœur (2000, 129) thus grants ‘unreservedly’ the possibility of ‘a phenomenology of feelings and dispositions that can be qualified as religious by virtue of the disproportion within the relation between call and response’. He also calls this disproportion an ‘affective fragility’, which is the major anthropological theme from his early work onwards. It is due to the fact that a response to a call cannot count on any prior and shared field of understanding as it would be the case with the correlation of question/answer. With the call thus a quite different articulation of responding breaks forth, namely one that centrally involves passivity, i.e. a ‘preethical obedience’ (*Gehorsam*) on the side of the one who hears (*hören*) or rather hearkens (*horchen*) the extraordinary height of the calling.^18^

The difficulty here does not concern the limited capacity of intentional consciousness to constitute a *beyond of intentionality* in terms of representation. As to Ricœur (2000, 129), it rather revolves around ‘the status of *immediacy* that could be claimed by the dispositions and feelings allied with the call-and-response structure in a religious order’. It is in this context that he declares the *inherently hermeneutic character* that a ‘phenomenology of religion’ has to assume. With this decision he definitely parts ways with the *radicalized phenomenologies* which revolve around the idea of some originary, unconditioned, or absolute *self*-*givenness*. Yet the necessity of running a hermeneutic detour is not simply derived from the fact that the dispositions related to the ‘basic feelings’ are embodied linguistically. Indeed, this problem had been discussed insufficiently by the early Husserl who considered language as an ‘unproductive layer’. Later, however, it has been embraced on various accounts, most exemplarily by Merleau-Ponty, the earlier Derrida, Richir, and also Ricœur. Yet the difficulty for Ricœur rather revolves around the fact that, as he puts it, ‘to the *linguistic* mediation a *cultural* and *historical* mediation is added, of which the former is a mere reflection’. Consequently, religion may only be considered ‘like language itself, which is realized only in different tongues’. This fact, however, ‘condemns phenomenology to run the gauntlet of a hermeneutic and more precisely of a *textual* or *scriptural* hermeneutic’ (2000, 130). The ‘continent of the religious’ thus appears only scattered and one cannot locate anywhere the universality of *the* religious phenomenon. The afore-mentioned feelings, e.g. nowhere appear in their ‘naked immediacy’ but are always already lived *as interpreted*.

As Ricœur consequently concludes, a ‘phenomenology of religion’ must renounce the task to approach the phenomenon in its ‘indivisible universality’. Furthermore he contends that ‘we must be content, at the outset [*sic*!], with tracing the broad hermeneutic strands of just one religion’. This implies, like Heidegger (2010, 89) emphasized in this context, to start ‘from out of our own historical situation and facticity’, and to consequently oppose the ‘claims of a rootless comprehension’ (Ricœur 2000). The only further option left thus concerns the idea of an ‘analogizing transfer’ that might, on the long run, concretize the regulative ‘*idea* of a phenomenology of religion as such’. To counteract the apparent restrictions of this project, Ricœur consequently appeals to an ‘interconfessional, interreligious hospitality’ that is designed to offer a medium of dialogue and change between the religions. This conception revolves around a posture of thought that accepts an ‘untranslatable kernel’ in each religion: according to Ricœur (2010, 31), recognizing this abyss paves the path for their mutually enriching exposure to the other, thus mobilizing the self-critical resources mobilized by the self-exposing ‘intelligence of faith’. Yet this undoubtedly rich idea – and its articulation in terms of the work of translation – is not the point I would like to get to.^19^ The critical point that interests me is a different one. It concerns the status of the *givenness* of the ‘absolute affection’ or the ‘immemorial gift’ that is all too quickly transposed into a hermeneutic discourse in this conception. As Ricœur himself realizes, the experiential quality of the extraordinary givenness that is at stake here and that can only give rise to its further articulations, becomes itself the ‘greatest enigma’ (Ricœur 2000, 132). This is the case since we cannot confront it beyond the circular hermeneutic status of the phenomenology of religion we are dealing with.^20^

However, as Kühn (2013, 353–355) has convincingly shown, Ricœur also appeals to instances of such ‘absolute givenness’ in his hermeneutics of religion. And indeed, without the pre-givenness of the absolute we would, in the last analysis, only know *texts*, i.e. interpretations of interpretations. Given this, the *attestation* of an unconditional and pre-narrative origin is absolutely central for Ricœur’s conception, since his idea of an ‘originary affirmation’ of something unconditional that cannot be experienced in the ‘bad infinity’ of reflection alone (i.e. in the condition of ‘existential difference’) definitely requires a pre-hermeneutic horizon of possible reconciliation (‘human mediation’) for the ‘broken cogito’.^21^ Since, however, a direct intuition of the absolute appears impossible for Ricœur, he has to watch out for ‘traces of the holy’ (ibid.) whose *attestation* opens a space of convergence (but definitely not identity) between the ‘hermeneutics of the absolute’ and the ‘originary affirmation’ that is experienced in the very act of reflection. This crucial point is emphasized explicitly in Ricœur’s (1980, 144) ‘The hermeneutics of testimony’:

The absolute declares itself here and now. In testimony there is an immediacy of the absolute without which there would be nothing to interpret. This immediacy functions as origin, as *initium,* on this side of which we can go no further. Beginning there, interpretation will be the endless mediation of this immediacy. But without it interpretation will forever be only an interpretation of interpretation.

Given this, the event of manifestation and its interpretation are constitutively and irreducibly *intertwined*. This assessment may definitely be true. But does it already provide us with sufficient reasons to start ‘directly from the manifestation of the world by the text and by scripture’, as Ricœur contends in ‘Toward a Hermeneutic of the Idea of Revelation’, that is, without delving any deeper into the *phenomenology of religious subjectivity and practice*? Ricœur (1980, 98) indeed mentions a proviso here:

This approach may seem overly limited due to the fact that it proceeds through the narrow defile of one cultural fact, the existence of written documents, and thus because it is limited to cultures which possess books, but it will seem less limited if we comprehend what enlargement of our experience of the world results from the existence of such documents. Moreover, by choosing this angle of attack, we immediately establish a correspondence with the fact that the claim of revealed speech reaches us today through writings to be interpreted.

I do not want to oppose this (theological) idea of the ‘process of the word’ directly. Yet I wonder whether the very ‘superabundance’ that it reflects and the ‘economy of the gift’ (Ricœur 1995, 315–329) that it is said to make accessible are not dependent in their very realization upon *inherently embodied experiences*. Wherein, we might ask, does the individual’s attempt to transform the overabundance of this lived *call* into the however coherent articulation of one’s concrete *response* find its concrete embodiment? As far as I see, this question concerns not so much the experiential (pre-)givenness of the *call* as its *responsive articulation and attestation*. Put differently, it concerns the phenomenological status of *experiencing interpretations of foundational experiences*, which, to my understanding, becomes reduced to a very specific and narrow interpretation of experience in this case.

This problem becomes prominent in the context of the claims that are negotiated between philosophical and theological hermeneutics. But such methodological questions are not important here. I am rather interested in the wider hermeneutic circles Ricœur deals with in this context, since they exactly serve to relate experience and interpretation in ever new form and depth. This regards firstly the circle conjoining the inspired word and the confessing community, a circle which gains its significance by way of establishing a ‘relation of mutual election […] between the one and the other’. Finally, as Ricœur (2000, 133, 135) holds, this circle is transposed into an ‘existential question’ on the level of the individual believer who is called, according to the contingency of his situation, to transform the ‘accident’ of his belonging to a community of faith into a continuously chosen destiny. This *existential twist* that the question of mediation receives on the level of the individual is, however, far from being an innocent move. Given that the links between personal and collective identity are passively woven and habitually functioning, thus displaying ever ‘fragile identities’ (Ricœur 2011), it is by far not only a *personal* wager with regard to a textual corpus of interpretation one may deliberately choose from. Concretely viewed, it rather relates back to one’s community as a ‘collective I-can’ and, hence, necessarily appeals to its ‘inter-corporeal semantics’, the ‘affective economies’ it entails and the patterns of performative enactment they prescribe. Thus viewed, a reconstruction of this situation by way of a *textual* hermeneutics involves both a far-reaching *dis*-*embodiment* as well as ‘*methodological individualization*’ *of the responding subject*. It is exactly this twofold deficit which needs to be countered on philosophical terrain in order to develop a genuine, i.e. integrated phenomenology of religion. How this can be conceived concretely and how it may help us to confront religious violence, will be discussed in the following section.

## 3. Some steps towards a phenomenology of religious violence

Our critical reassessment of the major contemporary phenomenologies of religion might be summed up as follows: contrary to first evidence, they do not deal with ‘lived religion’; thinking within a rarely explicitly avowed ‘framework of high culture’ (Ricœur 2010, 37),^22^ they rather either attempt to rethink God in terms of superabundant ‘givenness’ or focus a textual hermeneutics that translates such givenness into the narrative semantics of the respective religious system of knowledge. At a first glance, this criticism might appear as a too bold verdict. It is, however, strongly sustained by the fact that these positions largely focus on the analysis of *personal* religious experiences at the expense of its larger social, cultural and political dimensions, including its various institutions or embodiments (Mensch 2009). As a consequence, one need not wonder that the interested reader will not find any broader examination (not to speak of critical discussion) of religion in terms of an inherently ‘social’ phenomenon in the context of contemporary phenomenology of religion. Religion’s so-called ‘return’ and the issues it involves, including its assumed affinity to ‘irrational violence’, rather still appear as a kind of *terra incognita* for phenomenological research. This seems to be the case since it apparently threatens the teleological outlook of reason defended by phenomenology. Exactly the question, however, if phenomenology is able to conceive of a different brand of reason, that is, one that avoids the pitfalls of either an inherently ‘religious reason’ or the subjection of religion to the limits of ‘reason alone’, is essential and will need further treatment.

As regards the first option, we have observed in our discussion of Marion and Henry that the discourse on givenness – the ‘higher reason’ according to Marion (2008, 145–153) – all too easily runs the risk to turn the phenomenon into a ‘myth of the given’ and finally a *paternalism of givenness*, which is easily transformed into a ‘theology of grace’.^23^ Ricœur’s focus on the irreducible hermeneutic mediation of man’s openness to ‘the originary’ testifies to a strong sensibility for this difficulty that any ‘radicalized’ phenomenology of religion needs to confront. Ricœur, for his part, consequently convicted phenomenology to investigate the hermeneutic mediations of ‘the process of the Word’. That, however, he also appealed to the eventmentality of the absolute in his hermeneutics without explicitly framing it with his earlier practical-philosophical reflections on human affectivity, finitude, and ‘fallibility’, (Ricœur 1986) is but the other side of the coin. And indeed, any attempt to elaborate a truly integrated phenomenology of religion will need to bear this difficulty in mind: to prove the integrity of its subject, it needs to demonstrate how to avoid both the Scylla of the ‘all too originary’ that lacks evidential provability as well as the Charybdis of a ‘hermeneutics without end’ that threatens to dissolve the very phenomenon into a mere chain of interpretations.

But that is not all. To this difficulty another one has been added. By focusing either ‘counter-experiences’ of radical transcendence or the narrative structures of religious texts that symbolically substitute for an always already lost origin (‘Word’), these approaches remain limited also in another regard: not only the proponent of a ‘radicalized phenomenology’ but also the eminent hermeneutic phenomenologist of religion, both tend to eclipse or at least undervalue the constitutively bodily and inter-corporeal dimension of religious interaction. Put differently, they both do not deal with *embodied practices of making transcendence together*, that is, practices which revolve around the responsive articulation of the gift’s affective appeal in the practical horizon of narratively shaped identities and collective world-views. As a consequence, we need to not only take into account a *beyond of the text* that affects one personally and calls for the subject’s responsive articulation; we also need to consider the habitualized practices or *liturgies that concretely articulate this affective call by performatively translating it into the fabrics of everyday existence.* To put this in Schutz’s terms, the ‘paramount reality’ of the everyday life-world, which is ruled by the ‘pragmatic motive’, is not only left behind by taking the ‘religious attitude’ but is also ‘seen through’ (Schutz 1962, 257). Given this, however, the everyday life-world can be taken up, when viewed from the ‘finite province’ of religion, in a different light (grace, salvation, etc.), e.g. as a practical project of transformation, liberation, or possibly destruction. Put differently, I argue that an exploration of the *intertwining* between experience (affective call) and interpretation (response) in terms of embodied action will help us to develop a truly integrative phenomenological account of religion and, finally, religious violence. The related hypothesis thus is twofold: firstly, I argue that the *phenomenality of religion* unfolds nowhere but *in*-*between* experiences of ‘unconditional givenness’ and the autochthonous function of interpretation that is always already used to poetically integrate these limit phenomena into the course of everyday life; on the other hand, I hypothesize that our embodiment figures the *medium* of this encounter, providing the means to performatively translate the intelligibility of faith into the pragmatics of everydayness. In other words, I argue to search for the very phenomenon of religion – and henceforth religious violence – at the juncture where *inherently affective experiences of transcendence* and *embodied practices (or ‘liturgies’) of self*-*transcendence* intersect and, finally, even appear to merge.

Viewed against this backdrop, Ricœur’s conception of human existence as a ‘struggle for concordance in discordance’ receives a more dramatic twist; it does so once we realize that the intrinsically affective confrontation with the extraordinary – something that is habitualized in *rites de passage* and religious rituals – ‘offers a poetic license to start all over. To say it again. To do it again’ (Kearney 2006, 13). This primordial ‘moral creativity’ of human freedom to poetically refigure the ordinary, however, as Wall (2005, 52–53) remarks, is far from embodying an unanimously *good* power that only needs to be unleashed. Exactly in its radicality nests, as Kant’s reflection on ‘radical evil’ has anticipated convincingly (Ricœur 2010, 28–29), the irreducible possibility of violence. According to Ricœur’s basic anthropological insight, such ‘moral creativity’ as well as its other, both emanate from the ‘affective fragility of man’. Affectivity, to use a different terminology, exposes him to both impulses from below as well as from beyond – to e.g. drives as well as to a variety of ‘moral motivations’ (Ricœur 2010, 31) epitomized so famously (as well as under-determinedly) in Kant’s notion of *respect*.^24^

Perhaps Czech philosopher Jan Patočka (1989, 102) has recognized this basic ambivalence of religion in still unthought clarity:

This bringing into relation to responsibility, that is, to the domain of human authenticity and truth, is probably the kernel of the history of all religions. Religion is not the sacred, nor does it arise directly from the experience of sacral orgies and rites; rather, it is where the sacred qua demonic is being explicitly overcome. Sacral experiences pass over [to] religious as soon as there is the attempt to introduce responsibility into the sacred or to regulate the sacred thereby.

And as a sort of conclusion he furthermore adds: ‘religion is responsibility or it is nothing at all. Its history derives its sense entirely from the idea of a *passage* to responsibility.’ Responsibility here clearly is to be understood as a responsibility for the other – not only but, first and foremost, for *the other in myself*, that is, the disproportion that man’s ‘affective fragility’ entails. The aforementioned *passage* to responsibility thus is, as Derrida (1995, 35) comments, to be understood as a *project* that always has to confront the risk of submitting to a ‘return of the orgiastic’.^25^

As far as I see, ‘the orgiastic’ in Patočka and Derrida’s terms is equivalent to what the later Ricœur called ‘foundational excess of the source’. As to him, religious experience puts ‘capable man’, i.e. man who is capable of speaking, acting, narrating and, finally, of assuming moral responsibility for all these activities, at his most radical test. It does so since it not only confronts his ‘I can’ with an ‘I cannot’ that would testify to his factual finitude. It rather exposes his capacity to a both liberating and overburdening experience of the infinite, here understood in terms of a suffered infinition:

[D]welling in my finite capacity is something infinite, which I would call foundational. Schelling speaks of a *Grund*, a ground or foundation, which is at the same time an *Abgrund*, an abyss, therefore a groundless ground. Here the idea of a disproportion arises which is suffered and not simply acted upon, a disproportion between what I would call the excess of the foundation, the *Grund/Abgrund,* the groundless ground, and my finite capacity of reception, appropriation, and adaptation. (Ricœur 1999, 3; cf. 2010, 36)

On Ricœur’s account, the question of affectivity (suffering) is of course not dealt with in regard to the ‘history of religions’ like in Patočka’s afore-mentioned assessment. He rather focuses the problematic ‘confrontation between excess and moderation *within myself*’ and confesses that he ‘had not perceived for some time the *reserves of violence* implicated in this test of the finite capacity of the “I can”’ (Ricœur 1999, 4, my emphases), reserves that are best described by Bloch’s concept of ‘rebounding violence’.^26^ If for Ricœur the religious has as its function ‘the deliverance of the core of goodness from the bonds that hold it captive’; if it thus signifies ‘the capacity to make the ordinary person capable of doing the good’ (2010, 30), this does not exclude, as Wall (2005, 53) rightly stresses, ‘the tantalizing religious possibility that a *poetics* of the will (in the sense of describing the will’s Createdness) may imply also a poetics of the *will* (the will’s own capability for Creator-like Creativity)’. So even if the early Ricœur held that ‘to will is not to create’; that ‘human freedom’ inescapably finds its limits in a divine that it may ‘name’ only; and that ‘the creative source of the process of the release of goodness’ (Ricœur 2010, 34) can only be represented symbolically and mythologically – this is, as we know, not the true end of the story. And indeed, Ricœur at least rarely also glances at this other, deeply disconcerting possibility that is enshrined in man’s finite freedom, too: the possibility that man’s finite capacity of poetically liberating the original source translates into a mundane ‘gesture of transgression’ of everydayness, a gesture which hence cannot but run the *radical* risk to transform man’s irreducible disproportion into a ‘pretension to monopolize the source’ (Ricœur 2010, 35). It is here, in other words, that we touch upon the ‘strange possibility that humankind may be commanded to imitate its Creator by creating also (in however limited a way) its own human being in the world for itself’ (Wall 2005, 55).

Only once in his early work, in a discussion of the Church fathers, Ricœur faces this truly disconcerting possibility head on: ‘What should happen if [sc., like the fathers] we should invert the metaphor [sc., of being created in the image of God], if we should see the image of God not as an *imposed mark* but as the *striking power* of human creativity?’ (Ricœur 1965, 110; my emphasis) In all his work then, Ricœur discusses only the sense in which ‘naming God’ (1995, 217–235; cf. 262–275) implies an original kind of finite human ‘freedom’ that proceeds as a ‘poetics of the good’ (Ricœur 2010, 37) but more or less disregards the violent dimensions that also nest in man’s concrete capabilities for exercising such power. Whether this capability is related to – as in the Judeo-Christian tradition – man being created ‘in the image of God’ or not, the primordial binding that religion’s revolve around refers to ‘the possibility of receiving power from God [or whatsoever transcendent principle; M.S.] to overcome powerlessness’ (Kearney 2001, 108).^27^

Religious violence, thus viewed, can be identified as a sort of self-protective effort that seeks to contain the ‘excess of the foundation’ which, contrary to its inappropriable and excessive generosity, is appresented as being vulnerable to misperceptions and misinterpretations that appear to threaten the integrity of one’s religious identity. Or, as Ricœur (2010, 34–35) later puts this himself: ‘the creative source of the process of the release of goodness […] can be, as such, an object of mimetic rivalry’ which results in the ‘pretension to monopolize the source, to appropriate it in rivalry with the other recipient’s of the source fundamental generosity’. If, on this account, ‘fallible man’s’ finite capacities of reception cannot contain the foundational excess of a ‘groundless ground’ and can indeed never adequately respond to the superabundant logic of its call, this fact necessarily shapes ‘the receptacle with the partitions that fear and hate reinforce’ (Ricœur 2010, 36). Viewed against this backdrop, however, the poetic capability of man needs to be reconsidered according to its inherent ambiguity: if man’s capability grounds in the self’s irreducible tension between freedom and finitude, between the voluntary and the involuntary, it not only remains a ‘profound mystery’ which is not ‘knowable as part of any narrative identity’ (Wall 2005, 53); if the core of religion consists in exceeding our abilities to measure and contain the ground/gift and calls for an excessive practice of narrating, it rather may also translate in creating a narratively drafted world of yet unimagined possibilities for the believing self. Thus viewed, however, the wager that ‘the God who is named in the Bible [can] be experienced again in contemporary communities of interpretation’ (Wallace 1995, 50), may not only translate into liberating practices of textual deconstruction and re-imagination: it may also transform into a true ‘poetics of violence’ (Whitehead 2004), which revolves around the not only (while necessarily) destructive but also sense-making and self-transcending quality of such violent action. The inherently disorientating function of religious language may receive such a turn if man feels compelled to recover his own primordial humanity in face of the affective collapse of local communities (Ricœur 1998, 133) or the transformation of global ‘affective economies’ that disrupt long-distance empathy and inter-religious recognition, as in the case of our ‘new wars of religion’.

Indeed such possibilities of violent recovery are not unprecedented capacities. They rather already exist in manifold form as pre-given ‘scripts of action’ that can *contingently* be adapted to ‘define one’s situation’ accordingly, as Kippenberg has shown convincingly. In order not to shroud this ‘poetics of religious violence’ with the ‘nimbus of incomprehensibility’ and relegate it all too quickly to the realm of the irrational, we need to understand it in the *performative* terms of ‘communal religious action’ (Kippenberg 2011a, 1–18). Put differently, if, on the one hand, the ‘practical effects of religious worldviews and ethics depend on how believers frame their situation’, and if, on the other hand, ‘the frame they select determines the way they act’ (Kippenberg 2011b, 147), this intertwining of concrete action and its meaningful predisposition requires that we focus our attention to the *body* that is at stake in such kind of violence, figuring both its subject and object. This is indeed a necessary step, especially given the fact that authors like Kippenberg and Riesebrodt strongly emphasize the performative or interventionist (Riesebrodt 2007, 75–87) character of (violent) religious experience and practice that escapes both instrumental reason and normative motivations, but hardly deal with the inherently embodied nature of this subject.

The necessity to include a reflection on the body here becomes also graspable if we consider that the aforementioned intertwining between action and framing disposition can also be traced in our phenomenological terms of transcendence and self-transcendence. According to this same idea of intertwining, I proposed to analyze ‘lived religion’ to revolve around two foci: on the one side figures the experience of e.g. the holy, originary givenness, etc., that is, of something that irreducibly affects man and calls for response in terms of symbolic appresentation and interpretation; on the other side, we find interpretive cultures and ‘tales of transcendence’ which yet require the performative re-enactment of their experiential ground in order to ‘function’. Put differently, experiences of transcendence need to be translated into experiences of self-transcendence – the absolute needs to ‘declare itself here and now’, as Ricœur put it, and to this end has to ‘take flesh’. In its hybrid constitution, being both affectable and affecting, the body as a phenomenal or ‘lived body’ (*Leib*) is key in this translation: as ‘the most originally mine’ it is both the affective site of experiencing transcendence and, as a symbolic, habitual, and expressive body it is also a medium of self-transcendence.

Interestingly, the young Ricœur is also helpful in this context for grasping this both cardinal phenomenon in its basic ambivalence. In his ‘diagnostics’ of bodily expression in *The Voluntary and the Involuntary* he traced the intersections between affectivity and thought, a project that led him to interpret the ‘lived body’ as a body-knowledge, a savoir-faire, coming close to Simone Weil’s interpretation of the *ego cogito* in terms of an ‘I can’. Most importantly, Ricœur in this context did not simply adopt Merleau-Ponty’s basic insight into the body as a ‘vehicle of being-toward-the-world’ but proceeded further by exploring the *affective sensibility* of the body as a general, i.e. ‘affective medium’ for evaluation that makes possible the specifically human disclosure of the life-world:

[T]he fact is that the body is not only a value among others, but in some way involved in the apprehension of all motives and through them of all values. It is the affective *medium* of all value: a value can reach me only as dignifying a motive, and no motive can incline me if it does not *impress my sensibility.* I reach values through the vibrations of an affect. (Ricœur 1966, 122)

This important passage anticipates a genuine phenomenology of the ‘lived body’ that bears the potential to explore and spell out the intersections of affectivity, evaluation, and action. It points at, as Kearney (2015, 117–121) has demonstrated, the radical hermeneutic idea of a ‘carnal imagination’ that is able to disclose the silently functioning linkages between our embodied existence and an inherently inter-corporeal ‘poetics of the possible’. While Ricœur due to his ‘textual turn’ has not further elaborated on this line of thought, I see no reason why we should not link his late reassessment of the ‘lived body’ as ‘the mediator between the intimacy of the self and the externality of the world’ (Ricœur 1992, 322) with this earlier insight into the flesh as a general ‘affective medium’. In light of this, the re-inscription of Ricœur’s hermeneutic phenomenology into a social ontology of otherness in *Oneself as another* (which was realized with the help of Husserl, Levinas and Henry’s phenomenologies of embodied passivity) offers a template for *re*-*embodying and materializing his hermeneutics of religion*. To do so, we only need to include one further insight, originally formulated by Husserl and spelled out most clearly by Levinas, namely that the affective body is both active and passive; both vulnerable and capable of *transcending* this vulnerability: as Levinas (1979, 164) puts this, in it ‘sovereignty and […] submission […] are simultaneous’. The sovereignty of the ‘I’ qua embodied ‘I-can’ *is* also its vulnerability. The same counts for a collective ‘I can’, which disposes of socially derived ways of transcending the vulnerability of its embodied condition, but as an embodied ‘I can’ also remains tied to this condition. In its poetic capacity to transcend its situation, the ‘functioning body’ (Husserl) is ‘beyond sense’ (Mensch 2009, 78), non-substituable, and hence a truly ‘individuating facticity’ (Marion 2002a, 96–98); but this ‘functioning body’, this ‘most originally mine’ is also at once an ‘objective’, a ‘symbolic’ and an ‘expressive body’ that is vulnerable to physical forces as well as to socially overdetermined patterns of signification.

The importance of understanding embodiment in terms of intertwining becomes especially relevant in regard to religious violence, which revolves exactly around this ambiguity that the body embodies. Here the fact that the ‘lived body’ both figures man’s affective or animal part and her god-like openness to transcendence is central: it is only inasmuch as the affective body is ‘beyond sense’ that it can also figure as a ‘symbolic body’ and, thus, as a ‘medium of transcendence’. In this transcending character we encounter what can be called the *trans*-*religious sacrality of the body* that is grounded in its being ‘beyond sense’. The contingent articulation and unfolding of such ‘functioning sacrality’ includes practices of collective embodiment that exercise, train, and habitualise a community’s capabilities to experience the transcendent and let it exert an impact on everydayness. In their rebounding capacity to affectively ‘see through’ or transfigure everydayness and its pragmatic imperatives, these practices or liturgies symbolically (or rather carnally) appresent the transcendent. As we have pointed out, this appresentation transforms but partly also carries on the primordial violence that the affective encounter with the sacred, transcendent, etc. involves, thus becoming itself a kind of ‘a-semiotic communication’ (Srubar 2016) with the sacred, transcendent, etc. As Srubar demonstrates convincingly, this primordial violence is translated in a twofold way: on the one hand, it is worked into the semantic narratives or, to use Riesebrodt’s (2010, 85–86) terminology here again, ‘discursive practices’ of religious system of knowledge; on the other hand it becomes practically significant in a vast variety of ‘behavior-regulating practices’ that topologies of the profane and the sacred entail (Srubar 2016, 10–11). The violences pertaining to these narratives and the oftentimes rigid moral landscapes they engender, have frequently been noted and critiqued and remain on the radar of both religious and rational attempts to demystify religion. Today, the true philosophical quandary, however, is not to be found in these forms (this, however, does not exclude that they serve as a structural catalyst for those truly disconcerting forms of religious violence, as it happens, e.g. in the case of ‘suicide bombers’ when social histories of learned martyrdom transform into individual dramas of victimhood). We rather need to confront it in those excessively violent ‘interventionist practices’ or ‘liturgies’ that *today* performatively afford the religious, as Geertz (1973, 109) put it, with an unavoidable ‘aura of factuality’. Their specificity, as has been argued on various accounts across disciplines (Cavarero 2008; Nahoum-Grappe 2002; Salazar 2016, 129–131), derives from their ‘horrorist’ way of *desecrating* the other and, especially, the sacrality of her embodied being. *Desecration*, in Nahoum-Grappe’s (2002, 556) terms, is ‘the violation of the sacred’; it is the turning of the individual body’s being ‘beyond sense’ into a defaced, disfigured and abject ‘living object’ that hence not only suffers violence but more so suffers ‘shame before suffering: a purely sociological shame’ in face of the destruction of all social and personalized patterns of individual and collective identity.

On this account, the defilement of a grave or the destruction of religious sites in general, the rape of women, the enslavement of children, staged beheadings, mutilations, or the desecrating practice of torture ‘are therefore crimes of a similar nature in anthropological terms since they seek the same target lying at the very heart of the individual sense of sacredness’. The key point of course is that they not only attack this individual sense but also its social articulation:

A child or an old man, who may escape the grip of instrumental violence (which pursues an aim outside of itself), will not escape that of desecration since they are both, in their very bodies, emblematic links in the transmission of identity: one for its future promise and the other as proof of the roots that go back into the past. (Nahoum-Grappe 2002, 556)

The specific quality of such desecrating violence consists in the way it supplements violence with *cruelty*, thus rendering it capable to attack what escapes the destructive grip of instrumental violence. As far as I see it, a phenomenological analysis of this disparity offers a possible guiding-thread to confront and decipher inherently religious forms of violence, i.e. forms that are not simply qualified by the fact that religious semantics are conjured up to justify some otherwise instrumental, e.g. political violence (Cavanaugh 2009). In its poetic potential, such violence attempts to transgress the ordinary and everydayness, yet not in terms of its ‘coherent deformation’ within a ‘struggle for concordance in discordance’, but in the attempt to thus appropriate ‘the source of life itself’ (Ricœur 2010, 34, cf. 1999, 10). The problem with all this of course is that the ‘source’ here is thought in terms of ‘creative positivity’ alone, i.e. as if man were not able of ‘a primordial creativity ‘in the image of God – in the image, that is, of its own Creator’ (Wall 2005, 52). This, however, results in the problematic understanding that man’s role in the creation can be reduced to the idea of a ‘negation of negation’. As Sartre follows up in this context, this involves ‘a decision on human action’:

Everything positive being God, man by origin is negative (error, vices, crimes – all something negative). Therefore he is on God’s side not in creating the positive (which he cannot do since everything that can be already is), but in destroying the negative. By applying that negativity that is his own to destroy negativity. (Sartre 1992, 184)

Man in his attempt to ‘seize the source’ thus becomes, in Sartre’s terms, the ‘Anticreator’, dwelling in his attempts to negate a substantialized idea of negativity. This also explains why such violence not only intends the reinforcement of self-enclosure and the exclusion of the religious other (Ricœur 1999, 9–10) but necessarily involves inherently violent ways of self-determination, too, frequently shrouded in the projection of related self-hate onto its other. (Mensch 2011; Žižek 2008, 73) Furthermore, such violence absolves itself from all ends and transforms its means into ends in themselves, thus mimicking the habit of what might be called ‘divine violence’. Thus freed from any assumedly relativist moral economies as well as instrumental rationales, the ‘essence’ of religious violence tends to merge with its ‘performative’ (Kippenberg 2011b) and ‘phenomenal’ (de Vries 2013; Salazar 2016, 123) character. In its phenomenal display and performance, such violence – as a consequence to its ‘auto-immune reactivity’ – accepts an apparently unholy liaison with its very other, that is, scientific reason and its technological avatars. The related problem that we need to understand, as Derrida put it, concerns the fact that our ‘new wars of religion’ seem to take place in ‘two ages of violence’ at the same time:

In our ‘wars of religion’, violence has two ages. The one, already discussed above, appears ‘contemporary’, in sync or in step with the hypersophistication of military tele-technology – of ‘digital’ and cyberspaced culture. The other is a ‘new archaic violence’, if one can put it that way. It counters the first and everything it represents. Revenge. Resorting, in fact, to the same resources of mediatic power, it reverts (according to the return, the resource, the repristination and the law of internal and autoimmune reactivity we are trying to formalize here) as closely as possible to the body proper and to the premachinal living being. In any case, to its desire and to its phantasm. Revenge is taken against the decorporalizing and exproriating machine by resorting – reverting – to bare hands, to the sexual organs or to primitive tools, often to weapons other than firearms <*l’arme blanche*> […] This archaic and ostensibly more savage radicalization of ‘religious’ violence claims, in the name of ‘religion’, to allow the living community to rediscover its roots, its place, its body and its idiom intact (unscathed, safe, pure, proper). It spreads death and unleashes self-destruction in a desperate (auto-immune) gesture that attacks the blood of its own body: as though thereby to eradicate uprootedness and reappropriate the sacredness of life safe and sound. Double root, double uprootedness, double eradication. (Derrida 2002, 88–89)

What we see here attests to a reversal of ‘cause’ and ‘effect’ in the field of the religious: the phenomenal character of such violence apparently seeks to performatively reenact the ‘primordial violence’ that is, as I have argued, constitutive for the encounter with the transcendent. It attempts to do so by recovering or rather producing figures of ‘bare’, ‘unscathed’ or ‘sacred life’ by both the non-instrumental (sometimes indeed anti-instrumental) usage of cruelty and the destruction of its most ‘originary supplements’: tele-technology, technoscience, but also traditional cultures of interpretation that it reduces to their most literal meaning. Yet this very attempt of recovery at once cannot but participates in exactly these global cultural phenomena, thus unmasking a ‘deep interconnectedness […] of knowledge and faith, technoscience and religious belief, calculation and the sacrosanct’ (Derrida 2002, 90).

The fact that we need to confront this seemingly paradoxical ‘simultaneity of the incompossible’ (Merleau-Ponty) in order to think religious violence *today* is of paramount importance. But not only does a vast variety of such violence in its attempt to ‘seize the source’ use the means of technoscience and especially tele-technology in order to create evidence for the ‘aura of factuality’ that it aspires to re-create. As to this, let it suffice to mention the ‘porno-political’ proliferation of film footage covering beheadings and mutilations (Salazar 2016, 123–126), the excessive dissemination of jihadist ideology in the social media (Lohlker 2013), or the prominent case of the destruction of the Buddhas of Bamiyan by the Taliban regime, which has accordingly been analyzed as a first case of ‘performative iconoclasm’. Yet, this is, of course, not all. We also need to take into consideration an *inverse movement*, whose general structure became exceptionally clear in ‘the West’s’ response to the afore-mentioned destruction: this response revolved around the concept of ‘World cultural heritage’ and resulted in an auto-idolatrous sanctification of a Western ‘culture of reason’ which, at its centre, implemented a new category of transcendence – the transcendence of ‘works of art’ (Staudigl 2014). On a more general level, Derrida (2002, 63) in this same regard finally notes the following:

But inversely, if what is thus happening to us, as we said, often (but not always) assumes the figures of evil and of the worst in the unprecedented forms of an atrocious ‘war of religions’: the latter in turn does not always speak its name. Because it is not certain that in addition to or in face of the most spectacular and most barbarous crimes of certain ‘fundamentalisms’ (of the present or of the past), other over-armed forces are not also leading ‘wars of religion:’ albeit unavowed. Wars or military ‘interventions’, led by the Judaeo-Christian West in the name of the best causes (of international law, democracy, the sovereignty of peoples, of nations or of states, even of humanitarian imperatives), are they not also, from a certain side, wars of religion? The hypothesis would not necessarily be defamatory, nor even very original, except in the eyes of those who hasten to believe that all these just causes are not only secular but pure of all religiosity.

Religion, on this account, must not be understood as reason’s irrational and, finally, *evil other* but rather as its *accursed brother* and ‘censored chapter’. Given this, the positioning of philosophy vis-à-vis religion must not anymore be one that revolves around the assumption of a self-righteous philosophical logos, which is implemented to ‘purify’ factually existing religions, as if they were not always already ‘giving reason’ themselves (Marion 2008, 151). But it will also not anymore be the ideal of a ‘religion of reason’ – in a sense that stretches from Kant to Habermas – which is taken to unfold and articulate its cognitive potentials within the ‘bonds of reason alone’; we must not forget that this position definitely had (and still has) its problems to even think something like ‘religious difference’.^28^ If we manage to avoid these two positions we might finally, following the proposition by Jean Greisch (2004), hermeneutically approach a ‘third attitude’ that may prepare a different encounter of religion and philosophy – one that takes place under the sign of a reciprocal recognition of their respective alterity. This, however, is not anymore a hermeneutic endeavor in the traditional sense. Revolving around the cardinal insight into the irreducible role of the ‘lived body’ as the ‘affective medium’ of both transcendence and violence, it rather is a true challenge for a ‘carnal hermeneutics’ (Kearney) or what I prefer to call a diacritical phenomenology: its task, to which I sought to introductorily contribute here, is to phenomenologically recover ‘lived religion’ in-between a phenomenology of absolute givenness and a hermeneutic phenomenology of religious ‘texts’.

Phenomenology needs to face the task to confront ‘the disarray of the current “crisis”’ (*das Unwesen der gegenwärtigen Krise*), as Husserl (1970, 299) put it in his ‘Vienna lecture’ – the crisis now of *secularized* reason. Confronted with the novel realities of the ‘return of religion’, and especially ‘religious violence’, it needs to develop a heightened sensibility for a possible rebirth of reason: a rebirth, more precisely in response to a *pathos* that forbids us all nostalgic longing for reason’s assumedly possible *restitutio ad integrum*. In this sensibility thrives the promise of a *non*-*indifferent reason;* it would be one that does not shy away from confronting even the kind of violence that appears most irrational to it *on a par* with itself, that is, without self-righteously eclipsing its own violences – and be it by way of sacralizing its own claims. As Husserl (1970, 191) put it, it is definitely true also in this regard that ‘a one-sided rationality can certainly become an evil’.

## Disclosure statement

No potential conflict of interest was reported by the author.

## Funding

This article was conceived and written with the generous support of two research grants from the Austrian Science Fund (FWF). It was conceived in framework of the project ‘Religion beyond Myth and Enlightenment’ [grant number P 23255], and concluded in the project ‘Secularism and its Discontents. Toward a Phenomenology of Religious Violence’ [grant number P 29599].

## Notes

1. See, again Merleau-Ponty (1964, 178). This move indeed epitomizes the later Merleau-Ponty’s project that aims at a recovery of a ‘wild, vertical world’, or, to speak with Schelling, of a so-called ‘barbaric principle’ (Merleau-Ponty 1968, 267).

2. I take it for granted that Husserl’s lifelong venture consisted exactly in this: to defend the primacy of reason via a reflection of its ongoing and, perhaps, endemic crises. Reason, in other words, is something in the making for Husserl. So even his theory of a ‘primal institution’ (*Urstiftung*) is supplemented by his thoughts about a so-called ‘Nachstiftung’ and the necessity of ‘reactivation’, that is, as Merleau-Ponty (1968, 262) put it, on an unavoidable but creative practice of ‘coherent deformation’ that may only fulfill the destiny of reason in the history of its irreducible crises.

3. An instance of the enormous but frequently overlooked sensibility for such basic questions in Husserl can be found in his late ‘Vienna lecture’, when, for instance, he speaks about the ‘Unwesen der gegenwärtigen “Krise”’ (Husserl 1954, 347) an expression quite inadequately translated as ‘the disarray of the current crisis’ (Husserl 1970, 299). But indeed one would have to ask: a phenomenology of the *Unwesen*, of whatever *sein Unwesen treibt*, is it possible – and what would it look like? Isn't it exactly the never abandoned capacity for eidetic abstraction that figured so importantly in Husserl’s methodological treasure chest that is at stake once phenomenology encounters phenomena like violence (see Staudigl 2015) or religion?

4. A critical analysis of related topoi in Levinas’ work can be found e.g. in Bernasconi (1992), Sikka (1999), Ma (2008), and Maldano-Torres (2012). Recently, a more balanced reading has been provided by Blond (2016).

5. In the German original: ‘[Wir können uns] [n]ur auf der Grundlage einer selbstbewussten Verteidigung universalistischer Ansprüche […] von den Argumenten der anderen über unsere blinden Flecken im Verständnis und in der Anwendung der eigenen Prinzipien belehren lassen.’

6. In this context, Habermas’ argumentation revolves around the assumed "opacity" of religion and the related burden or ‘institutional proviso’ of translation (*Übersetzungsvorbehalt*): see Habermas 2008, 114–147.

7. This problematic is inherited from Kant whose political philosophy and philosophy of law is haunted by a deep ambivalence that revolves around the status of those who appear qualified as ‘reasonable subjects’. That this ‘qualification’ is dependent upon the historic origin and genesis of concrete human communities (and thus upon historic formations of founding violence) is definitely eclipsed by Kant’s (2009, 131) metaphysical deduction of law that abstracts from historic context in favor of an ideal of civil society.

8. What might concretely be derived from this insight for the further development of theological thought, would require further attention in itself but this is not the topic of this paper. As far as Levinas is concerned, see Purcell 2006.

9. This criticism is widespread. However, that Marion's conception of "givenness" can also be applied fruitfully to other religious traditions, is demonstrated convincingly by Schmuecker (2017) for the context of Buddhism.

10. Of paramount importance here is Schelling’s (1979, 212) break with the idea ‘dass das allem Vorauszusetzende […] auch das Vortrefflichste sein [müsse]’, i.e. his critique on the systematic conflation of the prior and the superior.

11. The latin *affectio* is derived from ad-ficere, which bears among others also the connotation of something being done to someone, perhaps violently.

12. Gondek and Tengelyi (2011, 189–206, 352–387) provide a much more positive interpretation at least of Marion, which defends his work against interpretations that see in it a deliberate transgression of the border between phenomenology and theology. However, as the discussion of love as a creative gift finally shows, Marion’s insistence on the ‘univocity of love’ finally subjects his ‘phenomenology of givenness’ to (an Augustinian) theological project (Gondek and Tengelyi 2011, 386–387).

13. The concept of ‘intergivenness’ (*interdonation*) which is essential in this context, is only mentioned at the end of *Being Given* (Marion 2002b, 323–324) and concrete analyses are lacking. Even the fact that Marion later provided, in *The Erotic Phenomenon*, a phenomenological analysis of love that in some sense takes this issue up, does not offer much relief since in its background still figures all too prominently a ‘*theo*-logy of love which leaves every theo-*logy* of love behind’ (Waldenfels 2012, 225).

14. Again, Gondek and Tengelyi (2011, 190–206) provide a different, much more positive interpretation of Marion. In this context, it concerns his reading of the correlation between call and response which, as they hold, results in a certain primacy of the latter that is neither contested by its belatedness nor by the excessive character and the anonymity of the call. Quite to the contrary, they rather understand his interpretation to attest to an irreducible historicity of the response, which may not only receive the call but may also *recall* it and, hence, abandon one’s constituting power to the benefit of letting givenness unfold itself (204–205). Marion (2002b, 306) himself talks about an ‘immanent decision’ on the part of the gifted which proceeds, as he emphasizes, ‘without vision or reason" since it concerns nothing but ‘the choice or the refusal of the great reason – of unconditioned givenness’. This, however, leads me to wonder whether phenomenology is not transformed here into metaphysics again, to be true not one to be understood in terms of ‘first philosophy’ but as dealing with its ‘last principle’ (Marion 2002a, 23–27).

15. Viewed against the background of my critique, the overall task definitely would consist in developing a *truly ‘responsive phenomenology’* of religion in Waldenfels’ sense. This author, unfortunately, has not dealt extensively with religion but has only offered some cues in this direction (Waldenfels 2012, 353–412). Interestingly, however, Waldenfels himself does not at all mention Ricœur in this context, who has provided, as will be demonstrated below, a phenomenological account of religion that strongly puts the focus on the responsive dimension, its articulations, and specific phenomenality.

16. As is well known, a ‘poetics of the will’ should have completed a trilogy among his early works, including *Fallible man*, which provided an ‘eidetics of will’ (Ricœur 1986), and *The Symbolism of Evil*, which offered exemplary insight into the ‘empirics of will’ (Ricœur 1969).

17. ‘We clearly reject the pretensions of an overly zealous apologetics which would pretend to derive God from nature or from subjectivity by a simple rational implication. / Thus we shall show rather the reverse impact of a philosophy of Transcendence (whose development we shall reserve for another work) on a philosophy of subjectivity. Our plan is limited to showing how, by starting with such a philosophy of Transcendence, philosophy of subjectivity is completed as a doctrine of *conciliation.* But by *showing* – rather than demonstrating – this completion, we are reading this philosophy of Transcendence, which erupts from above downward, in reverse. In reading it thus from the lower to the higher we shall discover the *response* of subjectivity to an appeal or a grasp which surpasses it’ (Ricœur 1966, 468–469).

18. This topic is dealt with early on by Ricœur (1974, 450–451, 466–467): in this appropriation of Heidegger's inaugural discussion of the ‘call of Being’ in terms of a ‘gift,’ the ‘word of God’ is rendered ‘existentially possible’ on the basis of this ‘preethical obedience’.

19. For a related exploration of the potentials of the concept of hospitality in the footsteps of Ricœur, Levinas and Derrida, see esp. Kearney 2011.

20. This of course refers to those circles embodied in the Jewish and Christian scriptures, that is, the ‘hermeneutic circles inherent to the scriptural constitution of the Jewish and Christian faith’ (Ricœur 2001, 132). See also the discussion below.

21. This famous triad of Ricœur’s philosophical anthropology, created with a view to Kant’s category of quality (reality, negation, limitation), i.e. the triad of *originating affirmation*, *existential difference*, and *human meditation*, is laid out explicitly in the conclusions of *Fallible man*, terminating in Ricœur’s (1966, 140) famous definition of man in terms of ‘the Joy of Yes in the sadness of the finite’..

22. Notwithstanding his focus on ‘banality’, this criticism also seems to be valid for Marion, given that his analysis of revelatory phenomena also unfolds against the backdrop of its articulation in such frameworks.

23. This is also Henaff’s (2010, 140, 418n.) critique of Marion (and even Derrida), which holds that in this position gift-giving is collapsed, at the cost of its ambiguously socializing capacity, into an inherently *ethical* figure.

24. At this juncture, it would be necessary to further investigate into the correlation between the ‘affective fragility of man’ and the notion of the ‘social bond’, a correlation that is regarded as absolutely constitutive in especially French social theory (Arppe 2014). Especially interesting for our topic of course is the fact that affectivity becomes a factor of social regulation exactly by way of its incorporation into a various ‘tales of transcendence’, including those that are ‘post-secular’ in nature (Giesen 2005).

25. This is a problem Patočka (1996) traced in his *Heretical Essays* in Platonism and Christianity’s attempts to incorporate the orgiastic – attempts that remain haunted by a respective suppression of affectivity or the orgiastic, which, hence, is always on the verge of returning. He also saw this same moment clearly, by the way, in the context of modernity and its discontents, epitomized in the experiences of the twentieth century, and especially with regard to the ‘First World War’, which he understands as a sort of collusion between the ‘return of the orgiastic’ and the hubris of technology in an unprecedented, nihilist atmosphere of ontological boredom. Upon closer examination, I would argue that his insight holds for the so-called ‘political religions’, too.

26. ‘Rebounding violence’, a concept coined by Bloch (2009), is useful for describing the ‘conquering return’ of the subject to this world from its ‘journey to the beyond’, (2009, 5) that is, to use a Schutzian expression, from the ‘religious finite province of meaning’ that has helped one to ‘see through’ the all too pragmatic limitations of everydayness.

27. The same argument can also be derived from Riesebrodt’s conception of ‘interventionist practices’ (Riesebrodt 2007, 75–87).

28. Kant’s seemingly funny polemic against the very concept of ‘religious difference’ in his late writing *On eternal peace* epitomizes this tendency; cf. Bernasconi 2009.
